# Mediation of the mediolateral ground reaction force profile to maintain straight running among unilateral transfemoral amputees

**DOI:** 10.1038/s41598-023-34288-4

**Published:** 2023-05-15

**Authors:** Ying Wai Tang, Akihiko Murai, Hiroaki Hobara

**Affiliations:** 1 Department of Human and Engineered Environmental Study, University of Tokyo, Kashiwa, Chiba 277-0882 Japan; 2 Human Augmentation Research Center, National Institute of Advanced Industrial Science and Technology (AIST), Kashiwa, Chiba 277-0882 Japan; 3 PRESTO, Japan Science and Technology Agency, Kawaguchi, Saitama 332-0012 Japan; 4 Faculty of Advanced Engineering, Tokyo University of Science, Tokyo, 125-8585 Japan

**Keywords:** Rehabilitation, Risk factors

## Abstract

The mediolateral ground reaction force (M-L GRF) profile that realizes a symmetrical mediolateral ground reaction impulse (M-L GRI) between both limbs is essential for maintaining a straight movement path. We aimed to examine the M-L GRF production across different running speeds in unilateral transfemoral amputees (TFA) to identify strategies for maintaining straight running. The average medial and lateral GRF, contact time (t_c_), M-L GRI, step width, and center of pressure angle (COPANG) were analyzed. Nine TFAs performed running trials at 100% speed on an instrumented treadmill. Trials were set at 30–80% speed with an increment of 10%. Seven steps from the unaffected and affected limbs were analyzed. Overall, the unaffected limbs exhibited a higher average medial GRF than the affected limbs. The M-L GRI were similar between both limbs at all speeds, implying that the participants were able to maintain a straight running path. The affected limb exhibited a longer t_c_ and a lower M-L GRF profile than the unaffected limb. The results showed that unilateral TFAs adopted limb-specific strategies to maintain a straight running path, and that these limb-specific strategies were consistent across different running speeds.

## Introduction

Over the years, the development of running-specific prostheses (RSPs) has tremendously improved the sporting performance of the individuals with lower extremities amputations^1^. A key function of RSPs is to allow users to regain their operational capabilities. Amputation surgery increases the risk of injury stemming from movement and locomotion in this population^2^. The annual fall rate for individuals with amputations ranges from 40 to 80%^2^. High fall rates have inevitably been attributed to a decrease in balance confidence and balance ability within the population, which reduces the desire to participate in physical activity^2^. Moreover, lower extremities injuries (both overuse and acute) are common among unilateral amputees from running^3^. Active participation in running in this population may increase the risk of physical injuries, degenerative joint disease due to overuse injuries, and lower back injuries due to compensatory strategies for the amputated lower extremities^4,5^. Therefore, it is important that research efforts to be directed at amputee locomotion and the development of RSPs to reduce the risk of injury and improve accessibility to sports for the population.

The ground reaction force (GRF) is often studied in relation to running mechanics because it is one of the main biomechanical variables that influences running velocity when examining human running mechanics^6^. The horizontal and vertical components of the GRF are often studied because they are the main factors controlling running velocity. Vertical GRF accelerates the body’s center of mass upward, whereas the vertical ground reaction impulse (GRI) controls the upward momentum^6^. These variables provide sufficient aerial time for leg swing during gait^7^. Similarly, the anterior–posterior GRF is responsible for the horizontal acceleration of the center of mass (COM), and the anterior–posterior GRI is responsible for controlling the horizontal momentum^7^. As the anterior–posterior GRF directly influences forward motion, it is mainly categorized into braking and propulsive forces. Sakata et al.^8^ found that despite having a similar vertical GRI, unilateral transfemoral amputees (TFAs) exerted a greater vertical GRF over a shorter contact time (t_c_) in the unaffected limb than in the affected limb. Makimoto et al.^9^ found that, among unilateral TFAs using RSPs, braking impulses were significantly smaller in the affected limbs than in the unaffected limbs. All these studies concluded that unilateral lower-extremity amputees had an asymmetrical force production profile between the affected and unaffected limbs during running.

On the other hand, the mediolateral GRF (M-L GRF) is rarely examined, as it tends to have a lower magnitude and higher variability than the horizontal and vertical GRF^6^. However, it is important to note that M-L GRF is related to the maintenance of balance and stability during straight running^10^. To maintain a straight movement direction, the net M-L GRI must be counterbalanced between the affected and unaffected limbs among the TFAs to maintain a net-zero M-L GRI during a stride cycle, which is defined as the period between two successive contact phases on the same foot^10^. The M-L GRI mediates the mediolateral momentum of the COM^11,12^. This should be counterbalanced between the two limbs to achieve a stable straight-path running. Specifically, unilateral TFAs would have to counterbalance the affected and unaffected limbs to maintain net-zero momentum. The inability to control running direction results in a higher risk of injury, which is often one of the main deterrents for the population to participate in physical activities and sports. In a study conducted by Hisano et al.^10^, they found that TFAs were able to maintain symmetrical M-L GRI using limb-specific strategy during walking. However, the mechanisms underlying the maintenance of a straight path among TFAs using RSPs remain unclear.

Step width (SW) is also a fundamental variable associated with the mediation of M-L GRF^13^. SW is defined as the mediolateral distance between two consecutive steps during walking. A previous study found that a wider SW was associated with a larger medial impulse and a smaller lateral impulse among able-bodied runners during sprinting^13^. However, these variables have not been thoroughly examined in patients with unilateral TFAs. Since SW was shown to influence the M-L GRI during the stance phase, examining the mediation of SW during running would provide valuable insights into how TFAs control their SW during running as part of their strategy to maintain a straight running path.

The center of pressure (COP) trajectory data showed a pattern of progression during the contact phase. The COP angle (COPANG) refers to the angle generated between the regression line of the COP trajectory curve and the intended movement direction. In a previous study, the COPANG was compared between normal gait and steppage walking gait among able-bodied individuals^14^. Steppage gait is defined as the inability to lift the foot while walking due to muscle weakness that causes dorsiflexion of the ankle joint^14^. It was shown that there was a significant difference between the COPANG exhibited from normal gait and steppage walking gait. Specifically, individuals with a steppage gait were shown to have a lower magnitude of COPANG than individuals with a normal gait. Generally, COP-related data are useful for evaluating the pathophysiology of individuals^15^. Therefore, COPANG could potentially be a measure of dynamic postural control for the maintenance of a straight movement path. To date, no study has examined the implications of COP angle and its relationship with the maintenance of movement direction, especially among TFAs, to identify strategies for maintaining a straight running path at various submaximal running speeds.

Therefore, the purpose of this study was to investigate M-L GRF development across different submaximal speeds among unilateral TFAs and to establish asymmetries in the running mechanics between the affected and unaffected limbs. The second objective was to examine the running strategy adopted by TFAs to maintain a straight running path. The first hypothesis was that the affected limb would have a different M-L GRF profile than the unaffected limb. Specifically, the affected limb exhibited a lower M-L GRF magnitude than the unaffected limb. A previous study showed that t_c_ was longer in affected limbs than in unaffected limbs during running^8^. However, they have achieved similar vertical GRI profiles^8^. Therefore, the second hypothesis was that despite the asymmetries between the affected and unaffected limbs, TFAs are able to maintain a symmetrical M-L GRI profile. Specifically, the affected limb exhibited a lower M-L GRF with a longer t_c_, whereas the unaffected limb exhibited a higher M-L GRF with a shorter t_c_. Furthermore, SW is associated with the mediation of M-L GRF during running^13^. In able-bodied runners, it tends to decrease as running speed increases^13^. This implies that different mechanical requirements are required to maintain different running speeds. Therefore, the third hypothesis is that unilateral TFAs adopt different biomechanical strategies to maintain a straight running path at different running speeds.

## Methods

### Participants

Nine runners who underwent unilateral transfemoral amputation were recruited for this study. Demographic data are shown in Table 1. All the participants had competitive 100 m sprinting experience in official sprinting races. They were also training regularly (1–6 days/week) at the time of data collection. Each participant used their own prescribed RSPs and prosthetic knee joints; the models are shown in Table 1. The category of the prosthesis were classified based on their level of stiffness. The higher category number signifies higher stiffness. This was to ensure that all the participants were well acclimatized to their RSPs and were comfortable using them to perform multiple running trials. All participants provided written informed consent before participating in the study. The research protocol was approved by the Institutional Review Board (Environment and Safety Headquarters, Safety Management Division, AIST) and was conducted in accordance with the Declaration of Helsinki.

**Table 1:** This table shows the demographic data, RSP model, prosthetic knee joint model, 100 m personal best record, and corresponding 100% speed for each participant.

Subject	Sex (M/F)	Age (years)	Height (m)	Mass (kg)	Amputated limb	RSP model	Prosthetic knee joint	100 m personal record (s)	100 m average speed (m/s)
1	M	26	1.75	66.0	Right	Sprinter 1E90 (cat.3)	3S80	14.08	7.10
2	M	17	1.77	84.0	Right	Sprinter 1E90 (cat.4)	3S80	14.45	6.92
3	F	29	1.64	62.3	Left	Runner 1E91 (cat.4)	3S80	14.61	6.84
4	M	26	1.71	63.3	Left	Runner 1E91 (cat.3)	3S80	16.02	6.24
5	M	24	1.60	60.0	Right	KATANA-β (hard)	3S80	16.13	6.20
6	M	54	1.70	65.8	Left	KATANA-β (medium)	3S80	16.25	6.15
7	M	23	1.68	55.7	Left	Sprinter 1E90 (cat.3)	3S80	16.81	5.95
8	F	19	1.56	58.9	Right	Runner 1E91 (cat.3)	3S80	16.86	5.93
9	M	38	1.61	63.5	Right	Runner 1E91 (cat.3)	3S80	23.00	4.35
Mean		28	1.67	64.4				16.47	6.19
SD		11	0.07	7.6				2.51	0.77

*RSP* running-specific prostheses, *SD* standard deviation, *cat* category.

### Experimental protocols

**Figure 1:**
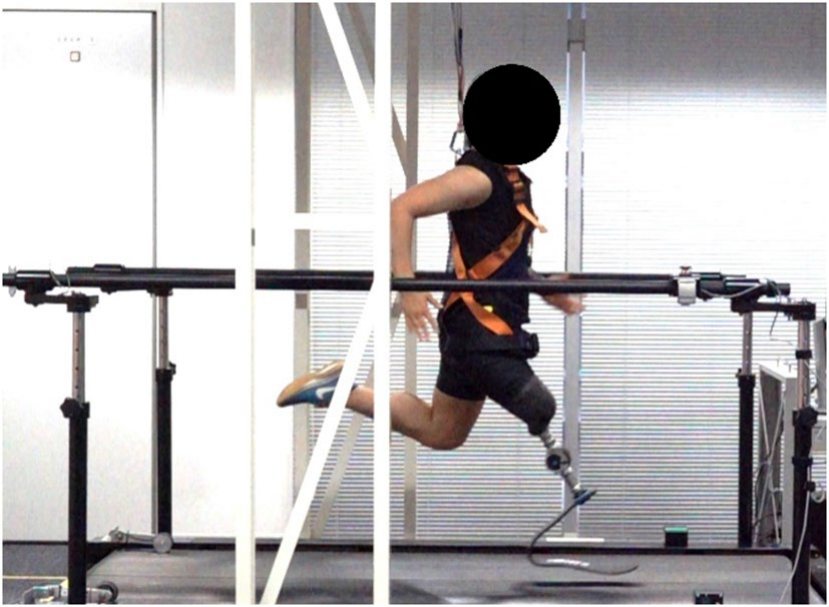
This figure shows the experimental setup. Two force plates were embedded within the split-belt treadmill. Nine subjects were instructed to run on the instrumented treadmill at six speeds (30%, 40%, 50%, 60%, 70%*,* and 80% of their 100-m personal best average speed. *A s*afety harness was donned on and handrails were readily accessible for the participants to ensure their safety.

All participants were given at least five minutes to familiarize themselves with running on an instrumented split-belt treadmill (FTMH-1244WA; Tec Gihan, Kyoto, Japan) before the running trials. Ultimately, all participants took more than 10 min. After completing the familiarization protocol, each participant was instructed to perform running trials on an instrumented treadmill at 100% speed (Fig. 1). In this study, 100% speed was defined as the fastest average speed (m/s) based on the participants’ fastest 100-m race time recorded in competitions. They were instructed to perform six trials in total, from 30 to 80% speed in increments of 10% for fifteen seconds each. To minimize the effects of fatigue, the participants were given ample rest time between each trial. The resting period between each trial was 1–2 min.

### Data collection and analysis

Two 6-df piezoelectric force plates (TF-40120-CL and TF-40120-CR; Tec Gihan, Kyoto, Japan) were embedded underneath each treadmill belt. These were used to collect GRF data with a sampling frequency of 1000 Hz. The GRF data were filtered using a low-pass Butterworth filter with a cutoff frequency of 25 Hz. From the filtered vertical GRF data, the touchdown and takeoff instants were detected using a threshold of 40 N^16–19^. This vertical threshold was used for all the GRF variables except the COP trajectory data. Owing to the sensitivity of the COP data, the vertical GRF thresholds for touchdown and takeoff were determined individually based on specific trials for each participant, ranging from 67 to 270 N. This threshold was determined by examining the first and last non-zero data points of both the COPx and COPy coordinates from the raw data. The zero data points can be understood as instances where the runners were in their flight phase, and the non-zero data points can be understood as the contact phase. Fourteen steps were analyzed in each speed trial for each participant. The fourteen steps were extracted from the middle of the running trials. This was to avoid any discrepancies in gait mechanics due to acceleration or deceleration. After identifying starting and ending the non-zero data point of each step, the highest corresponding vertical GRF was used as the threshold for that specific running trial and participant to determine the start and end of the contact phase.

**Figure 2:**
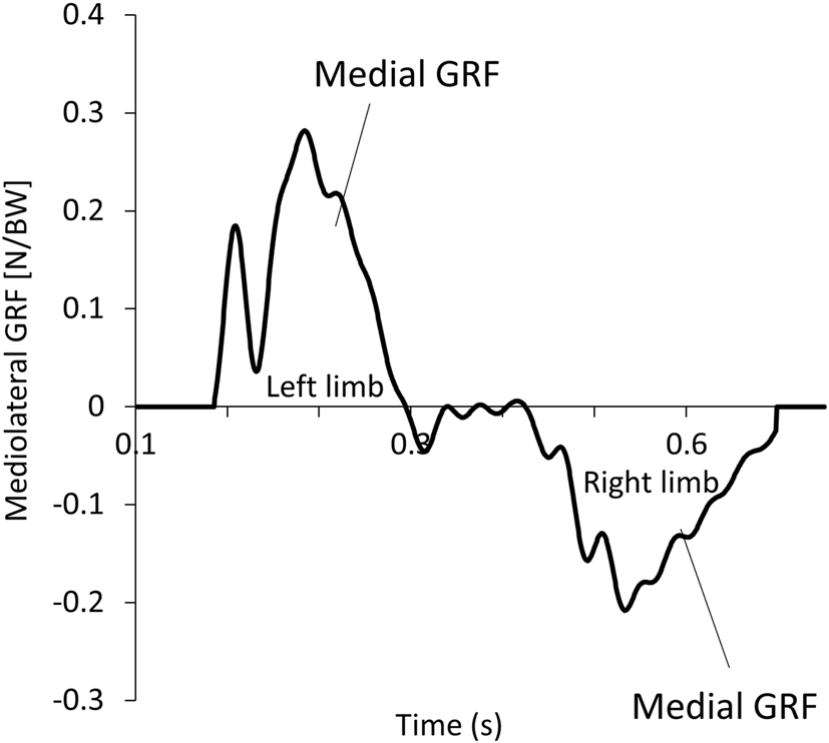
M-L GRF profile of two consecutive steps during running from one participant. M-L GRF, Mediolateral ground reaction force.

**Figure 3:**
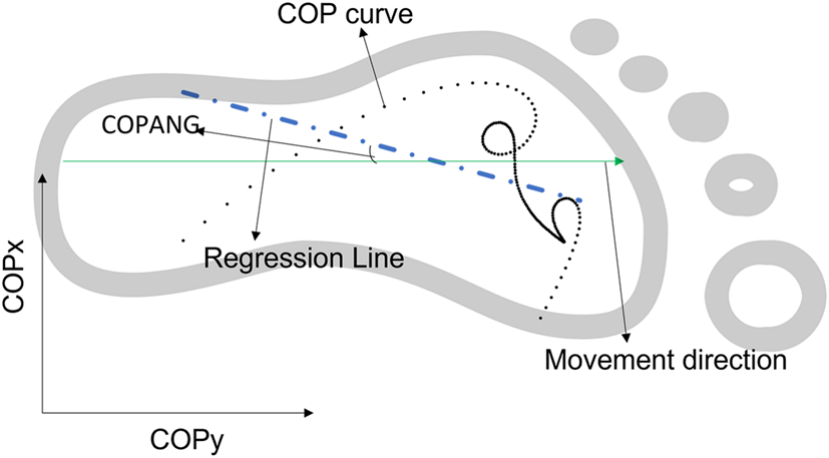
This figure illustrates the calculation of the COPANG from the COP trajectory data. COPANG, center of pressure angle; COP, center of pressure.

Owing to the nature of the production of the M-L GRF force, the directions of the M-L GRF production are bilaterally opposite. This is illustrated in Fig. 2. In the raw GRF data, the medial GRF of the left and right legs exhibit different polarities. For consistency, the numerical values of the medial and lateral GRFs were standardized as positive and negative, respectively. The step-to-step M-L GRI during the stance phase was calculated as the area under the M-L GRF curve using MATLAB Version9.12.0.1884302 (R2022a). SW is defined as the M-L distance between two consecutive COP positions that are representative of the foot positions during the contact phase. This was identified as the COP position at 50% of the stance phase at each step^13^. t_c_ was directly extracted from the raw data after touchdown, and toe-off instants were identified using a vertical threshold of 40 N, as previously mentioned. The COP data were also used to compute the COP trajectory data, which were subsequently used to compute the COP angle. The COP angle is defined as the angle generated between the linear regression line of the COP trajectory data and movement direction (Fig. 3)^14^. The pattern of progression of participants during running can be identified by tracking the path of the instantaneous COP during the stance phase. Because the COP data are representative of the COP position of the treadmill, the influence of the treadmill belt speed was removed as much as possible by adding the treadmill speed to the COP position data during the data analysis process so that the COP trajectory data were representative of the COP trajectories of the foot during the contact phase.

### Statistical analysis

Data distribution was checked using the Shapiro–Wilk test. If normality was confirmed (*p* > 0.05), a two-way repeated-measures ANOVA (speed [six levels: 30%, 40%, 50%, 60%, 70%, 80%] × limb (two levels: affected limb, unaffected limb]) was performed on the variables of interest to compare the variables between the limbs across the speeds. The assumption of variance was tested using the Mauchly’s test of sphericity. If the assumption of sphericity was violated, the Greenhouse–Geisser correction was performed to adjust the degree of freedom. Benjamini–Hochberg multiple comparison post hoc tests were performed if significant main effects or interactions were observed. If the normality of the data was violated (*p* < 0.05), the non-parametric Friedman test was used to compare differences. When a significant effect of speed or limb was observed, the Wilcoxon rank sum test with the Benjamini–Hochberg multiple comparison post-hoc test was used for post-hoc analyses. SPSS for Windows Version 26 was used, and statistical significance was set at *p* < 0.05.

## Results

The average running speeds for the respective trials were as follows: 1.85 ± 0.23 m/s (30%), 2.47 ± 0.31 m/s (40%), 3.08 ± 0.38 m/s (50%), 3.69 ± 0.46 m/s (60%), 4.31 ± 0.53 m/s (70%), and 4.92 ± 0.61 m/s (80%).

**Figure 4:**
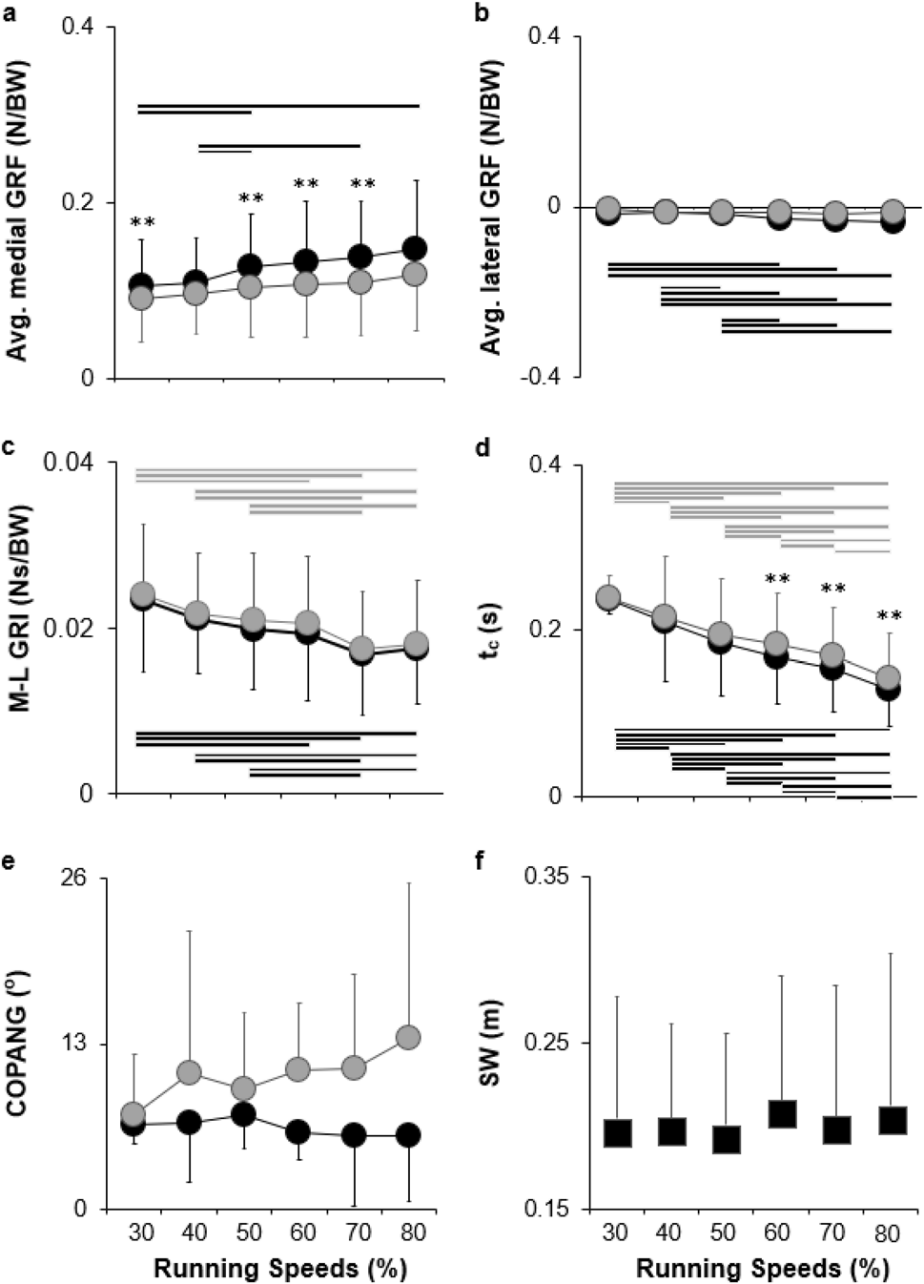
Results of all GRF related variables. Average medial GRF (**a**) & average lateral GRF (**b**), M-L GRI (**c**), t_c_ (**d**), COPANG (**e**), and SW (**f**) of the unaffected (black) and affected (gray circles) limbs across six different running speeds. Black (unaffected limb) and gray (affected limb) horizontal lines indicate significant differences at *p* < 0.05. GRF, ground reaction force.

As shown in Fig. 4a, significant effects of both limbs (*F*_(1, 8)_ = 10.39, *p* = 0.01, ES = 0.57) and speed (*F*_(5, 40)_ = 6.24, *p* < 0.01, ES = 0.44) were present on the average medial GRF. There was no significant interaction effect between the limbs and speed (*F*_(5, 40)_ = 1.53, *p* = 0.20, ES = 0.16). Post hoc tests showed that the average medial GRF of the unaffected limbs was significantly higher than that of the affected limbs in all running trials, except the 40% and 80% running speed trials. The results showed that the participants exhibited a higher average medial GRF in both the affected and unaffected limbs during the faster running speed trials. In addition, the unaffected limbs generally exhibited a significantly higher average medial GRF than the affected limbs.

Referring to Fig. 4b, the Friedman test showed a significant effect of speed on the average lateral GRF of the unaffected (*Χ*^2^_(5)_ = 28.18, *p* < 0.01) and affected limb (*Χ*^2^_(5)_ = 16.30, *p* < 0.01). However, the Wilcoxon rank-sum test and Benjamini–Hochberg multiple comparisons post-hoc tests showed that there were significant differences between speeds on the unaffected limb, but not on the affected limb. Specifically, the unaffected limb exhibits a higher average lateral GRF at higher running speeds. The Wilcoxon Signed Ranks test with Benjamini–Hochberg multiple comparisons post-hoc tests showed no significant differences between the affected and unaffected limbs across all running speeds.

Figure 4c shows that there was a significant effect of speed (*F*_(5, 40)_ = 7.95, *p* < 0.01, ES = 0.20), but no significant main effect of limb (*F*_(1, 8)_ = 72.03, *p* = 0.19, ES = 0.50) on M-L GRI. There was also no significant interaction effect between the limbs and speed (*F*_(5, 40)_ = 0.38, *p* = 0.86, ES = 0.05) on the M-L GRI. Participants exhibited a lower M-L GRI in the higher-speed trials than in the lower-speed trials. There were no significant differences in the M-L GRI across the 60%, 70%, and 80% speed trials.

The Friedman test showed a significant effect of speed on t_c_, unaffected (*Χ*^2^_(5)_ = 44.49, *p* < 0.01) and affected limbs (*Χ*^2^_(5)_ = 43.92, *p* < 0.01), which is illustrated in Fig. 4d. Both the limbs exhibited a lower t_c_ at higher running speeds. The Wilcoxon rank sum test also showed a significant difference in t_c_ between limbs at 60% (Z = − 2.31, *p* = 0.02), 70% (Z = − 1.13, *p* = 0.02), and 80% (Z = − 2.52, *p* = 0.01) speed trials. Specifically, the affected limbs exhibited a significantly longer t_c_ than the unaffected limbs in these trials.

Referring to Fig. 4e, the Friedman test showed no significant effect of speed on COPANG for unaffected (*Χ*^2^_(5)_ = 10.33, *p* = 0.07) and affected limbs (*Χ*^2^_(5)_ = 7.67, *p* = 0.18). The Wilcoxon rank-sum test with Benjamini–Hochberg multiple comparisons post-hoc tests showed no significant difference in COPANG between limbs across all trials. Referring to Fig. 4f, there was no significant effect of speed on SW (*Χ*^2^_(5)_ = 2.93, *p* = 0.71). The participants exhibited similar SW across all running speed trials.

## Discussion

This study aimed to investigate the M-L GRF profile during submaximal running in unilateral TFAs using RSPs. The results showed that the M-L GRF profiles were significantly different between the affected and unaffected limbs, which is in accordance with the first hypothesis and previous studies on unilateral TFA and transtibial amputee runners^7,20^. Specifically, unaffected limbs exhibited a higher average medial GRF than affected limbs. M-L GRFs are primarily mediated and modulated by ankle musculature, which includes the soleus, gastrocnemius, ankle evertors, and hip adductors and abductors^21^. Owing to the lack of a biological ankle joint in the affected limb, the ability to mediate the M-L GRF profile during the entire running cycle is restricted, which results in differences in the average medial GRF profile between the affected and unaffected limbs. In the current study, the average lateral GRF did not differ significantly between the affected and unaffected limbs. This was not in accordance with a previous study by Baum et al.^6^, which showed that the unaffected limb generated a significantly higher average lateral GRF than the affected limb across three speed trials (2.5, 3.0, 3.5 m/s). The difference in results likely stems from the difference in the running speeds tested. This study tested speed up to 4.92 ± 0.61 m/s. A higher running speed may have resulted in higher variability in medial and lateral GRF production. With a small magnitude, especially for the lateral GRF production, it was very sensitive to higher variability, which might be one of the reasons why no significant differences were observed between the unaffected and affected limbs in this study.

This study also showed that the t_c_ was generally longer in the affected limb than in the unaffected limb. This was particularly evident in trials with higher running speeds (60%, 70%, and 80%). This phenomenon was also highlighted in previous studies on unilateral TFAs during running and was suggested to be due to compensatory strategies adopted by this population^9,10,22^. Having a sufficiently high vertical GRI is essential to create sufficient aerial time to reposition the limbs in air to prepare for the next step^19,23,24^. In a study comparing faster and slower able-bodied runners^23^, similar vertical GRIs were achieved despite speed differences. The main distinction between the limbs was the ratio of the vertical GRF and t_c_ between these runners. In slower runners, the authors concluded that individuals had lower vertical GRF over longer contact periods^23^. Consequently, faster runners exhibited a higher vertical GRF over a shorter contact period. Owing to the restriction of force production in the affected limbs, they exhibited a longer t_c_ to allow for a lower GRF to be exerted over a longer contact period to achieve a similar vertical GRI between both limbs. Similarly, this phenomenon was evident in the M-L GRI profiles of the participants in this study. The M-L GRI is an important indicator of the ability of individuals to maintain a straight path movement^11^. Despite the asymmetrical M-L GRF development profile between the affected and unaffected limbs of the participants, a symmetrical M-L GRI profile was still realized by the amputees to maintain a straight running path. This is in accordance with our second hypothesis that TFAs can maintain a symmetrical M-L GRI profile despite asymmetries between the running mechanics of the affected and unaffected limbs. The calculation of the GRI is derived from the t_c_ and GRF. This study showed that the t_c_ of affected limbs was significantly higher than that of unaffected limbs. Simultaneously, the average M-L GRF was lower in the affected limb. The affected limbs exhibited a lower average M-L GRF over a longer contact period, whereas the unaffected limbs exhibited a higher average M-L GRF over a shorter contact period. This resulted in the limbs realizing a symmetrical M-L GRI profile across the running steps despite having a significant difference between the components that constitute the M-L GRI profile.

SW was not significantly different between the different speed trials in this study. This finding does not support the third hypothesis. Participants generally showed similar SWs across the different trials, despite speed differences. SW mediation is associated mainly with the frontal plane peak values of biomechanical variables among able-bodied individuals^25^. Specifically, a wider SW is associated with lower peak hip adduction and rearfoot eversion angles during walking^25^. Concurrently, a wider SW was associated with lower peak knee abduction moment and knee abduction impulse. Due to the loss of biological knee and ankle joints, it has been well established that there are significant strength deficits on the amputated side of TFAs^26^. Studies have also found that hip joint muscle strengths deficit in individuals with unilateral transfemoral amputations is up to 35% compared with able-bodied individuals^27,28^. Although hip abductors are not directly damaged by amputation surgery, it inevitably results in imbalances between the abductors and adductors^26^. Owing to these biomechanical restrictions, TFA runners compensate for their running strategy by having a relatively wider SW than able-bodied individuals to have lower peak frontal biomechanical variables regardless of running speed. Similar SW distances across different speed trials also reflect the inability of participants to mediate their SW during running.

It was also interesting to note that the M-L GRI decreased with speed and remained similar across the faster speed trials (60%, 70%, and 80%) for both limbs. This implies that there was a more than proportionate decrease in t_c_ and a less than proportionate increase in the M-L GRF profile, which explains the decreasing M-L GRI across the running speeds. The changes in the M–L GRF profile between faster and slower running speeds were not as profound as those in t_c_. In a previous study^9^, it was shown that the vertical GRI was not significantly different between different speed trials among TFA runners. As mentioned above, the vertical GRF and GRI are responsible for creating sufficient aerial time for limb swinging during gait. It is known that the vertical GRI must be sufficiently high enough in order to facilitate limb repositioning during limb swing^19,23^. One possible explanation for the similar vertical GRI across different running speeds is that the requirements for limb repositioning are similar across different running speeds. As for the M-L GRI, the differences in magnitude between the different speeds tested in this study may reflect the different requirements for maintaining a straight running path at different running speeds. This implied that the effort required to maintain a straight running path at a lower speed was higher. A similar M-L GRI at higher speed trials (60%, 70%, and 80%) also suggested that the effort required to maintain a straight running path would plateau at a certain point and would not increase despite an increase in running speed.

COPANG was found to have no significant differences across the different speeds for either the affected or unaffected limbs. There were also no significant differences between the limbs at different running speeds. However, it is worthy to note that the COPANG tended to be higher in the affected limb as compared to the unaffected limb. COPANG during locomotion is rarely studied in the existing literature. Jamshidi et al.^14^ established that the difference in COPANG between the normal gait and steppage during walking among able-bodied individuals was significant. The study showed that individuals with a normal gait exhibited a positive COPANG angle, whereas individuals with a steppage gait exhibited a negative COPANG angle. A positive COPANG angle represented a deviation from the direction of movement in the medial direction. A negative COPANG angle represented a deviation from the direction of movement in the lateral direction. However, the implications of the magnitude of COPANG have not yet been explored. COPANG is calculated as the angle between the movement direction and COP trajectory curves; therefore, this can be understood as the deviation angle from the straight-path movement direction during the contact phase. Despite the tendency for COPANG to differ between the affected and unaffected limbs at a faster running speed, participants exhibited a similar M-L GRI profile between limbs. This suggests that COPANG has little or no relationship with the maintenance of a straight running path.

In this study, TFAs achieved a net zero M-L GRI profile across multiple steps, rather than within a single contact phase. In a study conducted by Seethapathi and Srinivasan^29^, they tried to document step by step how able-bodied individuals correct for small imperfections in their running stride to avoid falling. They had concluded that able-bodied runners tend to exhibit a “one step dead-beat controller” on average for sideways velocity deviations, which is heavily associated with mediolateral impulse^29^. A “one-step dead-beat controller” essentially describes the phenomenon that able-bodied runners tend to mediate any changes in horizontal velocity within a single step. Although the implementation of this technique is not always perfectly executed, Seethapathi and Srinivasan^29^ concluded that there was a high tendency for able-bodied implementation of self-correction within a single step. This suggests that the mediation of the M-L GRF over multiple steps may be a unique strategy for amputee populations. It is speculated that the restricted ability to mediate SW may be the main factor behind this phenomenon. As previously mentioned, a relatively wide SW during running is associated with a larger medial GRI and a smaller lateral GRI^13^. Because of the wider SW, the TFAs were not able to counteract the medial GRI with the lateral GRI within one step. Therefore, the net medial impulse must be counterbalanced in subsequent steps. Future studies should be conducted to examine the differences in the M-L GRI profiles between able-bodied and TFA runners under similar conditions to determine whether the mediation of M-L GRI over multiple steps is a generalizable or unique strategy for maintaining a straight movement direction specific to different populations.

These findings can be applied to existing rehabilitation protocols and training programs for runners with unilateral TFA. The results of this study showed that maintaining a straight path might be more difficult at lower running speeds, as inferred from the higher M-L GRI value at slower speed trials. At lower running speeds, t_c_ was significantly longer than t_c_ at higher running speeds. This provides a longer duration for TFA runners to learn to maintain control as opposed to a faster running speed with a lower t_c._ In addition, TFAs would have to maintain knee extension on the affected limb for a longer period to prevent knee buckling with a longer t_c_. This is good training to build the necessary confidence and strength to serve as a foundation for running at faster speeds. Therefore, practitioners and RSP users should focus on low-speed running when trying to train for straight-running control. Training at a lower speed allows users to transfer this skill to a higher speed without the other risks that stem from running at a higher speed. In addition, the results of this study suggest that there are no differences in the effort required to maintain a straight running path beyond 60% of the maximal running speed. In relation to the maintenance of a straight running path, it is implied that there will be diminishing return training beyond 60% of the maximal running speed. This information can potentially be incorporated into training programs for TFA runners to enhance both performance and the ability to maintain a straight running path efficiently, focusing on training at slow-to mid-running paces.

Running performance is often simplified by examining the interaction between two spatiotemporal variables: step frequency and step length^30^. Specifically, the multiplication of these two variables provides the running speed. Higher step frequency and step length result in faster running performance. However, it is well known that there is a negative relationship between step length and step frequency^30,31^. Therefore, a more than proportionate increase in one variable and a less than proportionate decrease in the other is a common leverage that athletes use to improve their running performance. A lower t_c_ tends to result in a higher step frequency, and if there is less than a proportionate decrease in the step length, the running speed will increase. As suggested, a lower t_c_ reduces the window in which the running direction deviates from the intended direction. Additionally, a higher step frequency has been shown to reduce the magnitude of the GRF during loading, which is beneficial for alleviating the risk of bone and muscle overuse injuries^32^. Therefore, improving step frequency might holistically improve running performance, including the ability to maintain a straight movement path and faster running speed.

This study has a few key limitations. The RSPs and prosthetic knee joint models were not controlled for all participants. The participants were allowed to use their preferred models. Similarly, running shoes were not controlled. We decided not to control for the RSP model of RSPs due to the limitation of time for the participants to become accustomed to the designated RSP and prosthetic knee joint model. Controlling the model of the RSPs may negatively affect the running gait of the participants, and it is speculated that they may not be able to maintain the running direction as well as an unfamiliar RSP and prosthetic knee joint model. The objective of this study was to investigate the strategy that the population adopts to maintain a straight running path, and we aimed to implement the results in the general population with unilateral TFA, regardless of the RSP model. It is also important to note that the experimental protocol was performed using a treadmill. The running characteristics shown on a treadmill may not be fully representative of those shown during overground running^33,34^. Sinclair et al.^34^ found that lower-extremity kinematics were significantly different between overground and treadmill running among able-bodied runners, which is worth considering when discussing the generalizability of the results of this study.

## Conclusion

This study showed that unilateral TFA runners exhibited higher average medial GRF in the unaffected limb than in the affected limb. Despite these differences, they were able to maintain symmetrical M-L GRI profiles between the two limbs by adopting a limb-specific strategy. Specifically, the unaffected limbs exhibited a shorter t_c_ and with the combination of a generally higher M-L GRF, the M-L GRI generated was similar to that of the affected limbs, which had a longer t_c_ and a lower M-L GRF to achieve a similar M-L GRI between both limbs. At higher running speeds, the M-L GRI was significantly lower, suggesting that the maintenance of a straight running path is easier at lower running speeds. SW was similar despite speed differences, and COPANG of the unaffected limb showed no significant differences between limbs or across speeds. The general strategy for maintaining a straight running path was similar across the different submaximal running speeds. TFA also tends to achieve a net zero M-L GRI over multiple steps rather than within a single step.

## Data Availability

The datasets generated and/or analyzed in the current study are available from the corresponding author upon reasonable request.
